# Strengthening operational research to complement Global Fund grants for HIV, TB and malaria in Sierra Leone

**DOI:** 10.5588/pha.26.0007

**Published:** 2026-03-06

**Authors:** S. Kenneh, E.M. Kamau, A. Thorson, C. Halleux, G. Ameh

**Affiliations:** 1 Ministry of Health, Freetown, Sierra Leone;; 2 UNICEF, UNDP, World Bank, WHO Special Programme for Research and Training in Tropical Diseases (TDR), Geneva, Switzerland;; 3 World Health Organization Country Office, Freetown, Sierra Leone.

**Keywords:** tuberculosis, SORT IT, SDGs, universal health coverage, UHC

## Abstract

This issue of *Public Health Action* (*PHA*) includes the first of a series of articles that provide evidence-based answers to questions raised by health workers on addressing HIV, TB and malaria on the frontlines in Sierra Leone. The research was conducted as part of a SORT IT course, which is a partnership-based initiative led by TDR and implemented with various partners. SORT IT aims to make countries ‘data rich, information rich and action rich’ to improve health care delivery and outcomes. The course in Sierra Leone brought together 19 institutions to foster global engagement, partnerships and communities of practice, and highlighted the convening power of SORT IT towards galvanizing research capacity strengthening at the country level.

This issue of *Public Health Action* (PHA) includes the first of a series of articles that provide timely evidence-based answers to questions raised by health workers on addressing HIV, TB and malaria on the frontlines in Sierra Leone.^1-4^ Other articles in the series will appear in subsequent issues. The articles reflect the philosophy that:

‘All nations should become consumers and producers of research knowledge and research capacity needs to be strengthened, close to the supply of, and demand for health services.’ World Health Report 2013^5^

The series address the urgent need to accelerate progress toward the elimination of these diseases and achieving the Sustainable Development Goals (SDGs) by 2030.^6^ One of the requirements to achieving the health-related SDGs, including the control and elimination of infectious diseases of poverty (SDG 3.3), is to have robust programme data that informs strategies that improve access to care, enhance equity, and reduce mortality.^6^ SORT IT is a global partnership-based initiative led by TDR, the Special Programme for Research and Training in Tropical Diseases and implemented with various partners.^7^ It aims to make countries ‘data rich, information rich and action rich’ thereby improving health care delivery and outcomes.^8^ Scaled up to 97 countries, with over 1000 research projects, approximately 70% of SORT IT publications have shown an impact on policy and or practice.^9^ Evidence generated from such ‘real life’ settings is a form of public accountability and closely tied to achieving universal health coverage (UHC).^10^ SORT IT provides impetus by enhancing the availability of high quality, timely and disaggregated data for informed decision making. The expected impact is strengthened health systems, better programme performance and improved public health.

Building on the findings presented in this issue, and supported by previous SORT IT efforts,^11–13^ there is a renewed push for funding for operational research. From this perspective, Global-Fund supported programmes in Sierra Leone have begun incorporating operational research components into their national strategic plans and the upcoming Global Fund grant applications. By combining training with a hands-on approach, SORT IT participants are equipped to enhance program performance and strengthen national public health capacity, while cascading their knowledge through routine programmatic work. The current SORT IT course in Sierra Leone brought together 19 institutions from around the world, fostering global engagement, partnerships and communities of practice, while highlighting the convening power of SORT IT towards galvanizing research capacity strengthening at the country level. Three SORT IT champions capture the value and scope of this initiative:

‘This SORT IT initiative strengthens staff capacity, drives program impact, and generates local evidence to guide Sierra Leone’s HIV, TB, and Malaria strategies.’ S. Lakoh, Director of Disease Prevention and Control, Ministry of Health (MoH), and National SORT IT Mentor.

*‘*SORT IT has equipped me with key research skills and data-driven approaches, enabling our team to identify malaria hotspots and work more effectively toward elimination in Sierra Leone.’ A. Mac-Falama, Programme Manager, National Malaria Control Programme, MoH.

SORT IT has transformed my skills and strengthened Sierra Leone’s National Leprosy and TB Programme. It enhanced our research, proposal, and data-use capacity, enabling evidence-based advocacy, improved surveillance, and strategic decision making’ J.A. Koroma, Monitoring and Evaluation Officer, National Leprosy and TB Programme, MoH.
